# Characteristics of the ocular surface in neurotrophic keratitis induced by trigeminal nerve injury following neurosurgery

**DOI:** 10.1007/s10792-022-02521-0

**Published:** 2022-09-17

**Authors:** Caiyuan Xie, Bo Liu, Xiaoyu Zhao, Qing He, Lin Liu, Ruihua Wei

**Affiliations:** 1 Tianjin Key Laboratory of Retinal Functions and Diseases, Tianjin Branch of National Clinical Research Center for Ocular Disease, Eye Institute and School of Optometry, Tianjin Medical University Eye Hospital, Fukang Road, Nankai District, Tianjin, China; 2 Department of Neurosurgery, Tianjin Medical University General Hospital, Tianjin, China

**Keywords:** Neurotrophic keratitis, Neurosurgery, Corneal sensitivity, Tear film, Facial nerve paralysis

## Abstract

**Purpose:**

To analyse and quantify ocular surface parameters in patients with unilateral neurotrophic keratitis (NK) induced by trigeminal nerve injury post-neurosurgery.

**Methods:**

The study included 26 unilateral NK patients who had undergone neurosurgery, and 20 matched normal controls. Demographic and clinical characteristics of all participants were collected and analysed. Slit-lamp examination, Cochet–Bonnet aesthesiometry, Keratograph 5 M, and LipiView interferometer were performed on both eyes of 17 mild NK patients. For nine moderate/severe NK patients, sub-basal nerve density was measured by in vivo confocal microscopy.

**Results:**

Of the 26 patients, nine had acoustic neuroma, nine had trigeminal neuralgia, and eight had neoplasms. Facial nerve paralysis was observed in one of the 17 mild NK eyes (5.9%) and seven of the nine moderate/severe NK eyes (77.8%). Compared to contralateral and normal control eyes, 26 NK eyes showed significantly reduced sensitivity in five corneal regions (*P*** < **0.05). Corneal sensitivity in moderate/severe NK eyes was significantly lower than in mild NK eyes (*P*** < **0.05). Moderate/severe NK eyes had poor visual acuity, and their sub-basal nerve density was lower than that of the controls. The onset of the moderate/severe NK was from 0.5 to 24 months (median [Q1, Q3], 1 [0.5, 2.5] months) after neurosurgery. For the mild NK eyes, the number of total blinks, the first non-invasive tear breakup time (NITBUT) and average NITBUT were significantly lower than contralateral and normal control eyes (*P*** < **0.05), and the number of partial blinks and partial blinking rate were significantly higher than the other two control groups (*P*** < **0.05).

**Conclusions:**

Patients with NK induced by trigeminal nerve injury following neurosurgery had decreased corneal sensitivity to various degrees accompanied by increased partial blinks and shortened NITBUT. The severity of NK is related to the severity of the corneal sensory impairment. Facial nerve paralysis can worsen the clinical progression of NK.

Trial registration Chinese Clinical Trial Registry (ChiCTR2100044068, Date of Registration: March 9, 2021).

## Introduction

The cornea is one of the tissues with a dense innervation in the human body. Corneal sensory nerves originate predominantly from the ophthalmic branch of the fifth cranial nerve. To maintain a healthy ocular surface, corneal nerves release neurotrophic factors under basal physiological conditions [1] and tears provide growth factors and other nutrients after stimulation by the neurosecretory reflex [2]. The impairment of corneal sensory innervation causes a reduction in the vitality, metabolism, and mitosis of epithelial cells and the lacrimation reflex, with subsequent deficiency in epithelial repair and stromal and intracellular oedema [3].

The ophthalmic branch of the trigeminal nerve can regulate eyelid movements and the secretion of tears through the lacrimal, goblet cells and Meibomian glands by two reflex arcs (the motor and autonomic arcs) [4]. It plays a critical role in maintaining the integrity and proper function of the ocular surface [5]. Local or systemic diseases affecting anywhere between the trigeminal nucleus to the corneal nerve terminals may damage the trigeminal nerve [6]. Damage to the trigeminal nerve can lead to corneal hypoesthesia, further injury to the corneal epithelium, and the development of neurotrophic keratitis (NK) [7]. In the central nervous system, the treatment of trigeminal neuralgia by neurosurgical intervention is one of the most common causes of damage to the trigeminal nerve (post-surgical incidence of 2.8%) [8]. In addition, other common causes of NK are neoplasms, aneurysms, acoustic neuroma, or other surgical injuries to the trigeminal nerve [9]. Some neurosurgical procedures may simultaneously result in incomplete eye closure or exposure keratitis due to facial nerve (CN 7) injury [10, 11].

It is vital to thoroughly understand the mechanisms of the blink reflex when discussing trigeminal nerve damage combined with facial nerve damage. The corneal blink reflex is a loop between the facial nerve and the ophthalmic branch of the trigeminal nerve [12]. Previous studies found that decreased corneal sensitivity and decreased sub-basal nerve length were observed in all NK eyes [13, 14]. However, their studies were limited by the small number of patients. It is unknown whether the reduction in corneal sensitivity is directly associated with NK severity and it is unclear whether altered corneal sensitivity is restricted to the areas of nerve anomalies or all areas. It is useful practice to test sensations in the centre and in the peripheral four quadrants. The loss or decline of corneal sensation could decrease the sensitivity of the cornea to external stimuli, in turn, affecting the blink reflex. At present, there are few quantitative assessments of the corneal sensitivity and related parameters of blink and tear film stability in patients with different severity of NK. In this study, the severity of NK was graded according to the classification described by Dua [9]. NK was classified into three stages based on clinical signs: mild NK eyes presenting with epithelial and tear film changes without epithelial defects; moderate NK eyes presenting with epithelial defects but without stromal defects; and severe NK eyes presenting with stromal involvement ranging from corneal ulcer to lysis to perforation. This study looked at a population of patients with mild-to-severe NK caused by trigeminal nerve injury after neurosurgery, added corneal sensitivity as an important parameter for analysis. Additional indicators such as baseline characteristics, blinking parameters, tear film parameters and corneal nerve parameters were also assessed.

The aim of this study was to evaluate any differences in ocular surface characteristics between normal eyes, mild and moderate/severe NK eyes. It may help neurosurgeons and ophthalmologists identify potential risk factors early and allow them to intervene in a timely manner.

## Methods

### Patients

This study was conducted at the Tianjin Medical University Eye Hospital (Tianjin, China) between March 2021 and September 2021. Twenty-six patients with unilateral mild to severe NK who had undergone neurosurgery for acoustic neuroma, trigeminal neuralgia, or neoplasm were recruited from the Neurosurgery Department of a tertiary hospital. All operations were finished by the same set of experienced doctors. The unaffected contralateral eyes of the patients with NK and 20 eyes from normal subjects were analysed as controls. Patients with NK caused by diabetes, herpes virus, the wearing of contact lenses, eye surgery, or other types of keratitis were excluded. This study was approved by the Ethics Committee Review Board of Tianjin Medical University Eye Hospital and adhered to the tenets of the Declaration of Helsinki. Written informed consent was obtained from all patients.

### Ocular surface assessments

The diagnosis of NK was based on the history of trigeminal damage after neurosurgical interventions, which mainly showed ipsilateral corneal hypoaesthesia or anaesthesia. The severity of NK was graded according to the classification described by Dua [9].

**Fig. 1:**
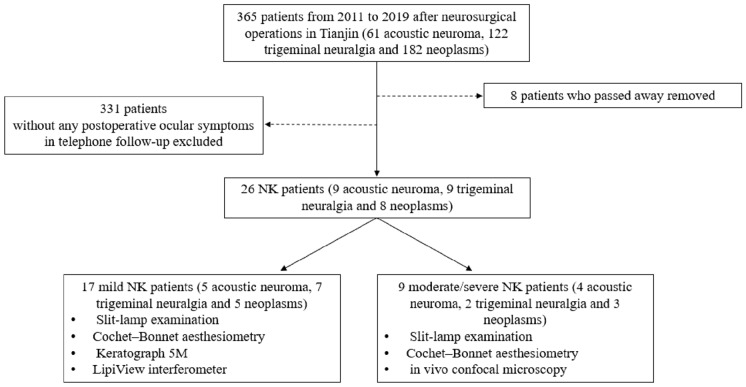
Flow chart of study selection process

All participants underwent best-corrected visual acuity (BCVA) and corneal sensation examinations. The BCVA was converted to the logarithm of the minimum angle of resolution (logMAR) value. Among these values, counting fingers (CF) before the eye was transformed as a logMAR value of 1.85 and light perception (LP) as a logMAR value of 2.7 [15]. In addition, Keratograph 5 M and LipiView were performed for both eyes in patients with mild NK and in healthy subjects. Patients with moderate/severe NK underwent sub-basal nerve density measurement using in vivo confocal microscopy (IVCM) (Fig. 1). For NK patients with the moderate/severe NK eyes, detailed clinical and demographic characteristics were also collected, including time from surgery, duration of NK, and so on. The "time from surgery" was noted as the time from date of surgery to most recent clinic visit. The "duration of NK" was defined as the days from time of surgery till time of diagnosis of NK.

Corneal sensation was evaluated bilaterally using a Cochet-Bonnet aesthesiometer (Luneau Ophthalmlogie, Chartres, France). This test applies different pressures to different regions of the cornea (superior, inferior, nasal, temporal, and central) using a retractable 6 cm monofilament nylon thread with a diameter of 0.12 mm. If a patient did not feel the filament at 60 mm, the filament length was reduced at 5 mm intervals until the filament was felt. The corneal threshold (inverse of sensitivity) is defined by the length of the longest filament necessary to obtain at least two positive responses in three stimulations.

Tear film function was evaluated using Keratograph 5 M (Oculus GmbH, Wetzlar, Germany). The first non-invasive tear breakup time (NITBUT-f) and the average non-invasive tear breakup time (NUTBUT-av) were described as the real time between the last complete blink and distortion of the placido rings. The tear meniscus height (TMH) and bulbar redness were also detected using Keratograph 5 M.

The LipiView interferometer (TearScience Inc., Morrisville, NC) can automatically evaluate lipid layer thickness (LLT) and partial blinking rate (PBR). A 20-s video documented the interference pattern of the tear film for each eye. We then analysed the interferometric colour units (ICUs) for each eye recorded in the video. One ICU reflects approximately 1 nm of LLT [16]. During the measurement, partial blinking and total blinking events were recorded, and the PBR was calculated as the rate between the two.

Corneal nerve measurements were performed using IVCM (Heidelberg Retinal Tomograph, Heidelberg, Germany; HRT II) from the central cornea corresponding to the site of assessment of sensation. Five non-overlapping images of the sub-basal nerve were randomly selected from the IVCM for nerve density calculation. Sub-basal corneal nerve density was defined as the total length of the nerves visible within an image frame (expressed in μm/mm^2^). We used ImageJ software (http://rsb.info.nih.gov/ij/) with the Neuron J plugin (http://www.imagescience.org/meijering/software/neuronj/) for corneal nerve semi-automated tracing and measurements.

### Statistical analysis

Categorical data are described as the number of cases and percentages. Fisher’s exact test was used in the presence of a sample size of less than 40. Data were examined for normality using the Shapiro–Wilk test. Normally distributed data were analysed with the mean ± standard deviation, the baseline comparison between the three groups was done using an ANOVA test and a two independent sample t-test was used to evaluate the differences between the two groups. For non-normally distributed data, the Mann–Whitney U test was used for two-group analyses. Data are presented as median (Q1, Q3). Statistical significance was set at *P*** < **0.05.

## Results

### Basal information

The basic information of all participants is listed in Table 1. There was no difference with respect to gender (*P* = 0.179) or age (*P* = 0.180) between the mild NK eyes, moderate/severe NK eyes and healthy control eyes. The underwent neurosurgeries of the 26 patients with NK included nine patients with acoustic neuroma, nine patients with trigeminal neuralgia, and eight patients with neoplasm (Fig. 1). Facial nerve paralysis was observed in one of the 17 mild NK eyes (5.9%) and seven of the nine moderate/severe NK eyes (77.8%). The incidence of facial nerve paralysis in moderate/severe NK eyes was significantly higher than in the mild NK eyes (*P*** < **0.001). Time from surgery in the moderate/severe NK eyes (23.8 ± 23.3 months) was significantly shorter than the mild NK eyes (49.9 ± 21.9 months) (*P* = 0.010). No clinical abnormalities were observed in the contralateral eyes of NK patients.

**Table 1:** Demographic data and baseline information of all participants

Affected eyes				
Characteristic	Mild NK eyes (17 eyes)	Moderate/Severe NK eyes (9 eyes)	Controls (20 eyes)	*P* value within groups
Ages (mean ± SD) (y)	60.8 ± 13.7	53.1 ± 8.9	61.8 ± 11.3	0.180
Male *n* (%)	7 (41.2)	6 (66.7)	6 (30.0)	0.179
Female *n* (%)	10 (58.8)	3 (33.3)	14 (70.0)	
Time from surgery (mean ± SD) (m)	49.9 ± 21.9	23.8 ± 23.3	–	0.010*
No facial nerve paralysis n (%)	16 (94.1)	2 (22.2)	–	< 0.001*
Facial nerve paralysis n (%)	1 (5.9)	7 (77.8)	–	

*NK,* neurotrophic keratitis; *y,* years; *m,* months. *P value < 0.05

### Outcome data and main results

For mild patients with NK, the corneal sensitivity in five corneal regions (superior, inferior, nasal, temporal, and central) was significantly lower than that in the contralateral eyes (*P* = 0.006, *P* = 0.006, *P*** < **0.001, *P*** < **0.001, *P* = 0.003, respectively), which was also significantly lower than that of normal control eyes (all *P*** < **0.001). Compared to contralateral and normal control eyes, the corneal sensitivity in these five corneal regions was significantly reduced in moderate/severe NK eyes (all *P*** < **0.001). Additionally, corneal sensitivity in the five corneal regions of moderate/severe NK eyes was significantly lower than that of the mild NK eyes (*P* = 0.003, *P* = 0.001, *P* = 0.002, *P* = 0.001, *P* = 0.001, respectively) (Table 2).

**Table 2:** Comparison of cornea sensitivity in mild to severe NK eyes with healthy contralateral eyes and control eyes

Cornea sensitivity (mm)					
NK Stages	Superior M (Q1, Q3)	Inferior M (Q1, Q3)	Nasal M (Q1, Q3)	Temporal M (Q1, Q3)	Central M (Q1, Q3)
Mild NK eyes (*n* = 17)	44.1 (35.0, 57.5)	43.5 (40.0, 55.0)	38.5 (25.0, 52.5)	42.1 (27.5, 55.0)	45.9 (47.5, 60.0)
Contralateral eyes (*n* = 17)	57.1 (55.0, 60.0)	56.8 (55.0, 60.0)	55.9 (55.0, 60.0)	57.9 (55.0, 60.0)	57.9 (55.0, 60.0)
Controls (*n* = 20)	58.3 (56.3, 60.0)	58.5 (60.0, 60.0)	60.0 (60.0, 60.0)	59.5 (60.0, 60.0)	58.8 (56.3, 60.0)
P1	0.006*	0.006*	< 0.001*	< 0.001*	0.003*
P2	< 0.001^#^	< 0.001^#^	< 0.001^#^	< 0.001^#^	< 0.001^#^
Moderate/Severe NK eyes (*n* = 9)	15.6 (5.0, 27.5)	7.8 (0.0, 10.0)	10.6 (0.0, 20.0)	10.0 (5.0, 12.5)	9.4 (0.0, 17.5)
Contralateral eyes (*n* = 9)	55.6 (55.0, 60.0)	56.7 (52.5, 60.0)	59.4 (60.0, 60.0)	58.3 (55.0, 60.0)	59.4 (60.0, 60.0)
Controls (*n* = 20)	58.3 (56.3, 60.0)	58.5 (60.0, 60.0)	60.0 (60.0, 60.0)	59.5 (60.0, 60.0)	58.8 (56.3, 60.0)
P1	< 0.001*	< 0.001*	< 0.001*	< 0.001*	< 0.001*
P2	< 0.001^#^	< 0.001^#^	< 0.001^#^	< 0.001^#^	< 0.001^#^
Mild NK eyes (*n* = 17)	44.1 (35.0, 57.5)	43.5 (40.0, 55.0)	38.5 (25.0, 52.5)	42.1 (27.5, 55.0)	45.9 (47.5, 60.0)
Moderate/Severe NK eyes (*n* = 9)	15.6 (5.0, 27.5)	7.8 (0.0, 10.0)	10.6 (0.0, 20.0)	10.0 (5.0, 12.5)	9.4 (0.0, 17.5)
P3	0.003^†^	0.001^†^	0.002^†^	0.001^†^	0.001^†^

*NK*, neurotrophic keratitis; *M*, Median; Q1, the first quartile; Q3, the third quartile; *mm*, millimetre

^*^P1 value < 0.05, *NK* eyes vs contralateral eyes; ^#^P2 value < 0.05, *NK* eyes vs controls; ^**†**^P3 value < 0.05, mild vs moderate/severe

**Fig. 2:**
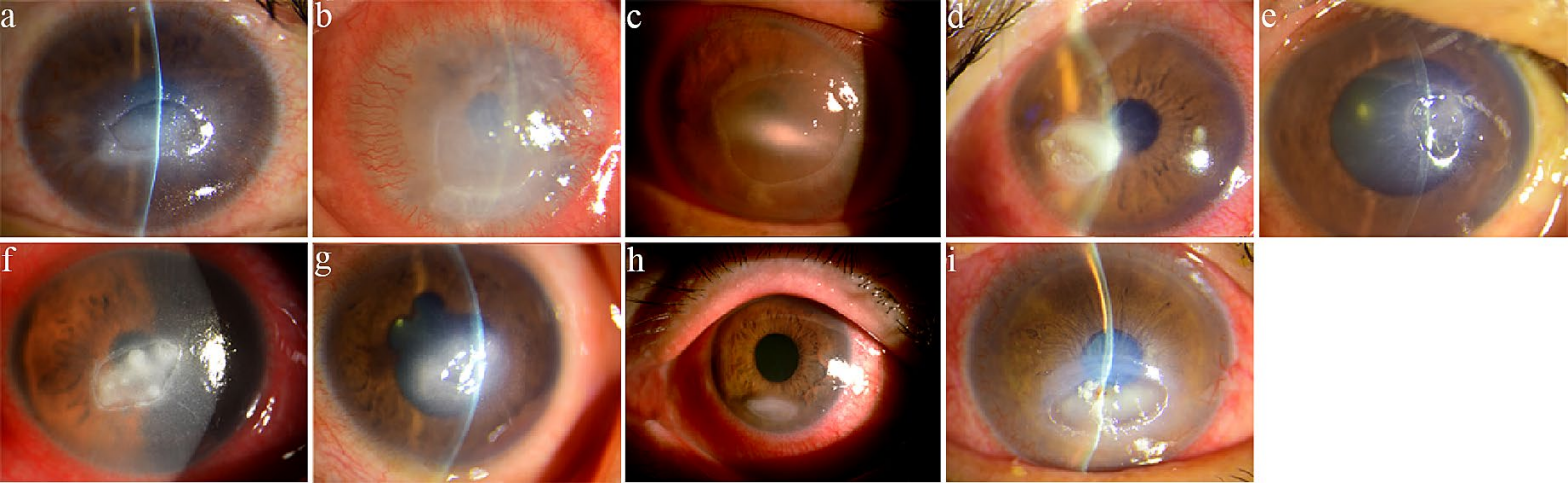
Representative anterior segment photographs of the nine patients with moderate/severe NK. The picture a–i are consistent with the No. 1–9 patients in Table 3. **a** The central cornea shows a diffuse haze with a large epithelial defect with elevated rolled edges. **b**The entire cornea is hazy with an epithelial defect with superficial and deep stromal vessels of limbus. **c** There is a large corneal epithelial defect with stromal opacities. The white, rolled margins of the defects is a typical sign of a non-healing epithelial defect. **d** Note the temporal persistent epithelial defect. The denuded surface appears dry, milky and hazy. **e** There is a large corneal epithelial defect with Descemet membrane folds. **f** Cornea epithelial defect with dense stromal infiltration on the centre cornea. It is surrounded by a slightly raised grey ring of proliferating epithelium. **g** Around the epithelial defect exists poorly adherent opaque and oedematous epithelium. **h** Superficial corneal ulcers located on the inferior side of the cornea. **i** A deep corneal ulcer with a small perforation was noticed, the perforation was inferior to the centre of the cornea and was 0.5 mm × 0.5 mm

**Fig. 3:**
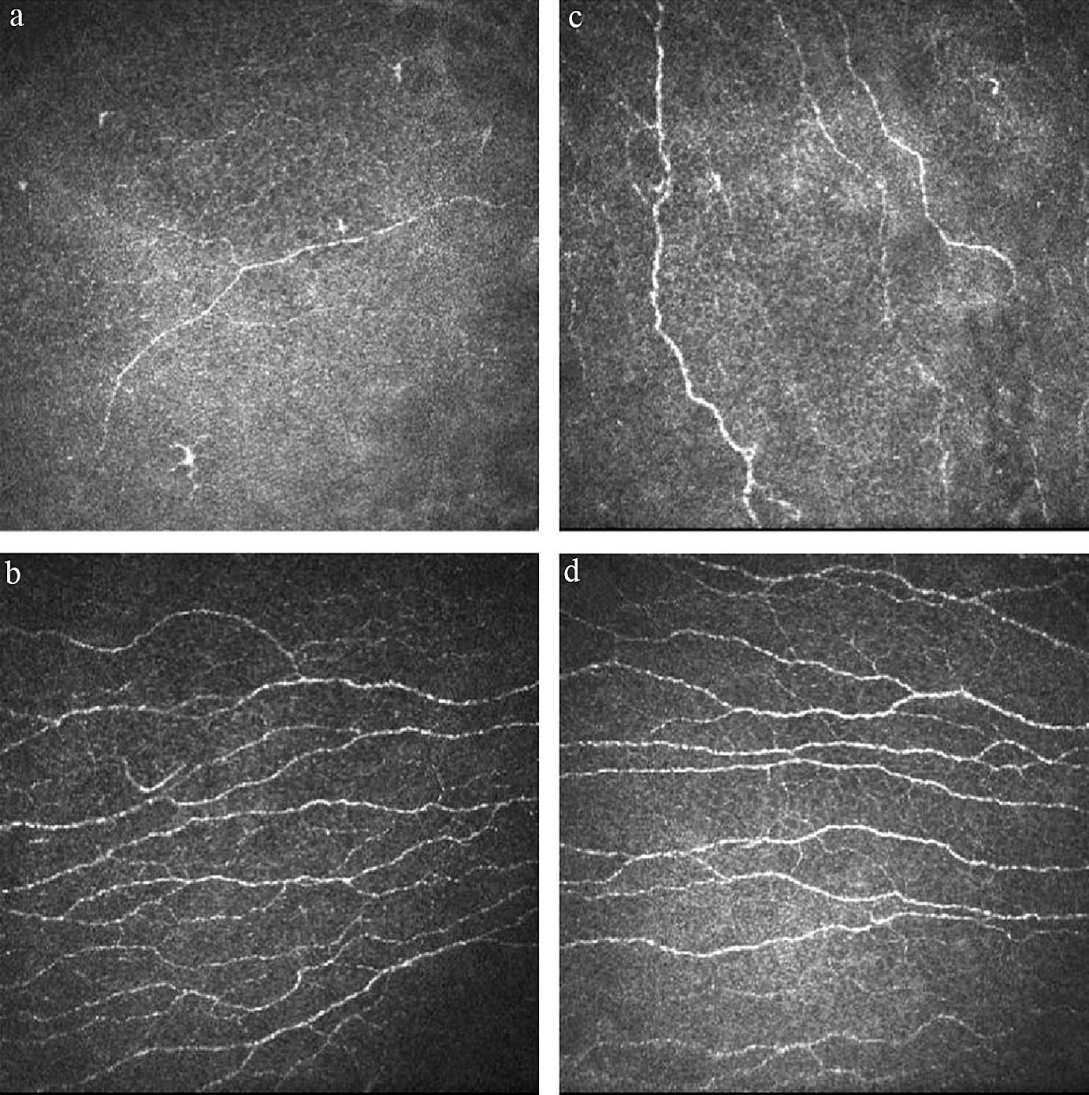
Corneal confocal microscopy images showing a reduction in the sub-basal nerve plexus (white lines) in moderate/severe neurotrophic keratitis eyes (**a**,** c**) compared with contralateral unaffected eyes (**b**,** d**)

Representative anterior segment photographs of the nine patients with moderate/severe NK are shown in Fig. 2. At the time of inclusion, all patients showed monolateral NK with persistent epithelial defects and stromal involvement ranging from corneal ulcer, lysis or perforation. Patients underwent neurosurgery in the past 2 to 72 months (median [Q1, Q3], 24 [3, 36] months) and were diagnosed as NK from 0.5 to 24 months (median [Q1, Q3], 1 [0.5, 2.5] months) after neurosurgery. The visual acuities (logMAR) were 1.38 ± 0.74 in nine moderate/severe NK eyes. The corneal sub-basal nerve densities were 5013.89 ± 2720.11 µm/mm^2^ in nine moderate/severe NK eyes (Fig. 3). Seven of the nine patients had damage of the seventh cranial nerve. Three (42.9%) patients mainly presented with facial palsy, three (42.9%) with lagophthalmos, one (14.3%) with lower eyelid ectropion, and one (14.3%) with ptosis. All moderate/severe patients had been treated with traditional therapy, including surgery and medication. One (14.3%) of those patients improved relatively fast but two (22.2%) patients recurred. The course of the disease varied from 3 to 16 months. Five (55.6%) patients were still under observation and others were lost to follow‐up. The improved corneas are presented as corneal nebula and corneal macula (Table 3).

**Table 3:** Clinical and demographic characteristics of the nine patients with moderate to severe NK eyes included in the study

Disease for neurosurgery (*n* = 9)									
Characteristic	Acoustic neuroma (*n* = 4)			Trigeminal neuralgia (*n* = 2)			Neoplasm (*n* = 3)		
Patient No	1	2	3	4	5	6	7	8	9
Sex	Female	Male	Male	Female	Male	Male	Male	Male	Female
Age (y)	50	62	52	52	71	39	49	51	52
Time from surgery (m)	3	2	72	14	48	24	24	3	24
Duration of NK (m)	3	2	1	0.5	24	0.5	1	1	0.5
NK stages	Moderate	Severe	Moderate	Severe	Moderate	Severe	Moderate	Moderate	Severe
BCVA (Log MAR)	0.7	2	1.85	1	0.6	0.7	1	1.85	2.7
CCS (mm)	0	0	0	0	5	0	25	0	0
Corneal nerve density (µm/mm^2^)	5188	1963	7830	1539	8414	3720	8720	4254	3497
Ipsilateral facial nerve involvement	Facial palsy + Lagophthalmos	Lagophthalmos	Facial palsy	Lagophthalmos	Lower eyelid ectropion	None	None	Ptosis	Facial palsy
NK treatments	Tarsorrhaphy	Autologous	Tarsorrhaphy	Ophthalmic	Ophthalmic	Autologous	Ophthalmic	CL	CT +
	+ CL	serum + CL		ointment	ointment	serum	ointment		Tarsorrhaphy

NK, neurotrophic keratitis; BCVA, best-corrected visual acuity; CCS, central cornea sensitivity; CL, contact lens; CT, conjunctiva transplantation; CF, count finger; LP, light perception; mm, millimetre; µm, micrometre; y, years; m, months

A comparison of the ocular surface parameters in 17 mild NK eyes, 17 healthy contralateral eyes and 20 control eyes is shown in Table 4. For the mild NK eyes, the number of total blinks was significantly lower than contralateral and normal control eyes (*P* = 0.004 and *P*** < **0.001, respectively). The number of partial blinks was significantly higher than the other two control groups (*P* = 0.037, *P* = 0.044, respectively). Similarly, the PBR was also significantly higher than the other two control groups (*P*** < **0.001, *P*** < **0.001, respectively). The NITBUT-f and NITBUT-av in the mild NK eyes were significantly lower than the other two control groups (NITBUT-f: both *P*** < **0.001; NITBUT-av: *P* = 0.002, *P*** < **0.001, respectively). The BCVA and other ocular surface parameters, including LLT, bulbar redness, and TMH in mild NK eyes did not differ significantly from those in the other two control groups (*P* > 0.05).

**Table 4:** Comparison of ocular surface parameters in mild NK eyes with healthy contralateral eyes and control eyes

Characteristic	NK eyes (*n* = 17)	Contralateral eyes (*n* = 17)	Controls (*n* = 20)	P1	P2
	Mean ± SD	Mean ± SD	Mean ± SD		
BCVA (Log MAR)	0.22 ± 0.17	0.15 ± 0.13	0.19 ± 0.18	0.206	0.471
LLT (nm)	75.47 ± 25.36	78.71 ± 21.20	73.20 ± 21.60	0.689	0.770
PBR	0.79 ± 0.29	0.28 ± 0.25	0.21 ± 0.23	< 0.001*	< 0.001^#^
Number of partial blinks	4.59 ± 3.41	2.41 ± 2.32	2.75 ± 1.83	0.037*	0.044^#^
Number of total blinks	5.76 ± 3.40	9.53 ± 4.11	11.95 ± 4.68	0.004*	< 0.001^#^
Bulbar redness	1.16 ± 0.34	1.08 ± 0.23	1.02 ± 0.22	0.378	0.114
TMH (mm)	0.22 ± 0.11	0.24 ± 0.09	0.25 ± 0.07	0.481	0.287
NITBUT-f (s)	4.00 ± 1.46	7.39 ± 2.29	7.42 ± 2.89	< 0.001*	< 0.001^#^
NITBUT-av (s)	6.65 ± 2.93	10.40 ± 3.62	10.84 ± 2.16	0.002*	< 0.001^#^

*NK*, neurotrophic keratitis; *BCVA*, best-corrected visual acuity; *LLT*, lipid layer thickness; *PBR*, partial blinking rate; TMH, tear meniscus height; NITBUT-f, the first non-invasive tear breakup time; NITBUT-av, the average non-invasive tear breakup time; mm, millimetre; nm, nanometre

^*^P1 value < 0.05, *NK* eyes vs contralateral eyes; ^**#**^P2 value < 0.05, *NK* eyes vs controls

## Discussion

NK is a rare corneal disease that occurs owing to partial or total impairment of the trigeminal nerve or its ophthalmic branch, leading to a reduction or loss of corneal sensitivity [3]. Due to the site and character of the lesions, trigeminal nerve damage is commonly found in patients with neoplasms, trigeminal neuralgia, acoustic neuroma [9], and so on. The compression brought about by neoplasms and surgical injury could cause some level of trigeminal dysfunction, leading to untoward side effects from sensory loss to the face, eye, or both. Dua et al. [9] proposed a new grading standard for NK, which would be more clinically relevant than the former standard and would indicate severity and prognosis of the disease. This study was designed to evaluate the differences in ocular surface characteristics between normal eyes, mild and moderate/severe NK eyes, in an attempt to find potential risks and carry out timely intervention.

Our results showed that seventeen of the patients with NK had mild cases. The results were inconsistent with previous reports where grade II ulcers were reported to be the most seen [17]. The possible reason for these inconsistent results is that all patients in this study came from post-neurosurgery follow-up by telephone. At the time of the telephone follow-up, all patients were willing to undergo a comprehensive ophthalmic examination. In previous studies, patients with NK were enroled mostly after seeking medical treatment in outpatient clinics. In this study, we have found that there were no apparent differences in healthy controls, mild and moderate/severe NK groups with regards to age or gender. For patients with NK, the symptoms of pain and discomfort may be less or absent due to hypoaesthesia or anaesthesia of the cornea. Most patients with NK visit the hospital after symptoms of visual impairment appear upon significant involvement of the central cornea. Consequently, the moderate/severe NK patients could be diagnosed after neurosurgery relatively early. Facial nerve damage typically occurs post-surgery to remedy acoustic neuromas, neoplasms, or globus jugulare tumours [11]. It can decrease innervation to the orbicularis oculi, which can result in lagophthalmos (incomplete eye closure) and even lead to excessive corneal exposure [10]. This study found that 5.9% of patients with mild NK had facial nerve paralysis, while occurring in 77.8% of patients with moderate/severe NK. The results illustrate that facial nerve paralysis can exacerbate corneal damage, resulting in worse outcomes.

NK is one of the most difficult and challenging ocular diseases. One of the reasons for this is decreased corneal sensitivity. Cochet-Bonnet aesthesiometry is the preferred method for older children and adults [18]. Kanpolat et al. [19] reviewed the surgical experience of 1600 idiopathic trigeminal neuralgia patients treated with percutaneous radiofrequency rhizotomy. Corneal reflex was absent in 5.7% of the patients, and keratitis developed in 0.6% of the patients. Mulhern et al. [20]. reported that corneal sensation decreased by 41.6% and sensation was absent in 8.4% of 62 patients who underwent surgery for acoustic neuroma. Patients with hypoaesthetic corneas had a higher incidence of corneal diseases (79%) than those with normal corneal sensitivity (39%). Hsu & Modi [17] reported a retrospective analysis of 20 patients with NK, in which there were no correlations between the severity of ulceration or degree of hypoaesthesia with visual recovery or outcome. This study found that corneal sensitivity was significantly reduced in NK eyes. Corneal sensitivity in moderate/severe NK eyes was significantly lower than that in mild NK eyes. It showed that the more severe the corneal perception injury, the higher the chance of disease progression. We speculated that the reason for the difference between the two studies was that the characteristics of the included patient population, such as disease severity and inclusion access, were different. Nonetheless, we were unable to draw specific conclusions on a disease-specific corneal sensation threshold below which neurotrophic ulcers can occur. The patients with NK in our study showed different degrees of decreased corneal sensation. When the condition progresses, it often escapes timely detection. Therefore, these patients were recommended to be monitored closely.

The prognosis of NK depends on the severity of trigeminal nerve impairment and whether it is accompanied by exposure keratitis [4]. In this study, we found that sub-basal nerve density in patients with moderate/severe NK was remarkably lower than healthy patients mentioned in the literature [21–23]. Previous studies using laser scanning confocal microscopes (LSCM) reported that sub-basal nerve density values were above 20 mm/mm^2^ in normal subjects [21, 23]. Reviewing the literature on the pathogenesis of NK, Müller et al. [5] suggested that epithelial cell metabolic activity and neuropeptides such as substance-P or vasointestinal protein will decrease after corneal denervation. Therefore, neurotrophic deficits play an indispensable role in the pathological processes of NK [24]. We also found that the visual acuities (logMAR) in the moderate/severe NK eyes were significantly lower than the mild NK and the normal control eyes. There was a vision screening of older adults conducted in the United Kingdom, if visual acuities (logMAR) with usual spectacle correction did not improve to less than 0.5, the person may be referred to an ophthalmologist [25]. Significant difference compared to 0.5 LogMAR may mean that the ocular surface of the moderate/severe NK eyes was in great danger because of a dual nerve injury. Early intervention is recommended if problems are detected immediately after neurosurgery. Recovering corneal sensation and treating exposed corneas simultaneously are critical for maintaining ocular health and preventing irreversible vision loss. Currently, the treatment for the NK was according to the severity of the corneal injury. The mild corneal epithelial changes are treated with preservative free artificial tears and ointments; the epithelial defects are treated with therapeutic bandage contact lenses, serum tears, amniotic membrane transplant and surgical tarsorrhaphy or chemically induced upper eyelid ptosis; the melted or perforated cornea are treated with conjunctiva transplantation, amniotic membrane transplantation and so on [26]. The traditional approach to treating NK has been supportive rather than addressing the underlying cause. There were several new treatments, such as various growth factors and neuropeptides (i.e. insulin-related growth factor 1, Substance P peptide, Cenegermin, Nicergoline) [7, 9, 26–29] and corneal neurotization [30–32] have been shown to promote corneal nerve regeneration and restoration of corneal sensitivity. A further study into these fields will help provide a better alternative for this difficult to treat condition.

The blink reflex is regulated by the loop between the facial and trigeminal sensory nerves. When the cornea is stimulated by external stimuli, the information is transferred to the brainstem via the ophthalmic division of the trigeminal nerve. It then projects to the facial nerve and activates the orbicularis oculi muscle, resulting in muscle contraction and subsequent blink reflex [33]. Disruption of this important neural circuitry may contribute to exhibiting NK. Therefore, all patients with trigeminal nerve damage should have their blink reflex evaluated to determine the effect of reduced corneal perception on stimulus delivery. The mean blink rate was 12–15 blinks/minute in humans, and was significantly higher in women than in men [34]. Blink rate is obviously decreased in patients with bilateral NK, while blink rate can be normal in unilateral cases because the other unaffected eye elicits normal symmetrical blinks [35]. The present study found using a LipiView interferometer that the number of total blinks in unilateral NK eyes was significantly lower and the number of partial blinks was significantly higher than that in normal eyes. A possible reason is that some of the blinks were not identified and recorded by the instrument because of an amplitude deficit. Previous research has indicated potential associations between incomplete blinking and reduced tear film stability [36]. This study found that partial blink increased but total blink, and NITBUT decreased in NK eyes. A reduction in the capability of blinking can result in a failure to homogeneously distribute tears over the ocular surface, destabilisation of the lipid layer of the tear film and increased excess evaporation. This can lead to secondary dryness and exposure [37]. For patients with mild NK, we recommend utilising artificial tears without preservatives and correcting blinking habits. Because the degree of corneal injury was slight, there was no significant visual decline in patients with mild NK. Furthermore, tear secretion and LLT in NK eyes showed no significant changes. It is possible that the decreased corneal sensation was still not sufficient to alter tear secretion. This finding is contrary to previous studies [14, 38] which suggested that tear production might be reduced consequent to a lack of reflex tearing from corneal irritation in NK.

This study had a variety of limitations. Firstly, this study included monolateral NK patients with three categories surgically induced trigeminal damage, the strength of this study was limited by a cross-sectional study. A larger study with long-term follow-up should be performed. Secondly, different variables were collected for each group and not for all in this study. This was because the moderate/severe NK eyes were not able to undergo Keratograph 5 M and LipiView examinations due to severe ocular surface insults. And, because the mild NK patients exhibited various degrees of decreased corneal sensitivity, the mild NK eyes were not examined by confocal microscopy in order to avoid non-healing cornea damage. Thirdly, central corneal nerve density can vary from one provider to another, and, because this study lacked control confocal, the comparison with the values in the literature may not be completely valid.

In conclusion, the study quantitatively analysed the ocular features of patients with mild to severe NK for the first time. Corneal sensitivity was significantly lower in moderate/severe NK eyes than in mild NK eyes and was often accompanied by facial nerve paralysis. The present study found that the BUT decreased, whilst PBR increased in mild NK eyes. Clinical optimization of blinking and the use of artificial tears may ameliorate NK symptom and benefit NK patients. It is of utmost significance for the long-term prognosis of patients with NK to evaluate indicators related to disease progression and to intervene promptly.

## Data Availability

The raw data supporting the conclusions of this article will be made available by the authors, without undue reservation.
